# Health At Every Size intervention® under real-world conditions: the rights and wrongs of program implementation

**DOI:** 10.1080/21642850.2022.2128357

**Published:** 2022-09-30

**Authors:** Audrey Cloutier-Bergeron, Amélie Samson, Véronique Provencher, Lyne Mongeau, Marie-Claude Paquette, Mylène Turcotte, Catherine Bégin

**Affiliations:** a School of Psychology, Université Laval, Québec, Canada; b Centre de recherche Nutrition, santé et société (NUTRISS), INAF, Université Laval, Québec, Canada; c School of Nutrition, Université Laval, Québec, Canada; d School of Public Health, Université de Montréal, Montréal, Canada; e Institut national de santé publique du Québec, Québec, Canada; f Department of Nutrition, Université de Montréal, Montréal, Canada; g Ministère de la santé et des services sociaux, Québec, Canada

**Keywords:** Program implementation, program outcomes, health at every size, participant responsiveness, adaptation

## Abstract

Implementation integrity is known to be critical to the success of interventions. The Health At Every Size® (HAES®) approach is deemed to be a sustainable intervention on weight-related issues. However, no study in the field has yet investigated the effects of implementation on outcomes in a real-world setting.

**Objective:**

This study aims to explore to what extent does implementation integrity moderate program outcomes across multiple sites.

**Methods:**

One hundred sixty-two women nested in 21 health facilities across the province of Québec (Canada) were part of a HAES® intervention and completed questionnaires at baseline and after the intervention. Participant responsiveness (e.g. home practice completion) along with other implementation dimensions (dosage, adherence, adaptations) and providers’ characteristics (*n *= 45) were assessed using a mix of qualitative and quantitative data analysis. Adaptations to the program curriculum were categorized as either acceptable or unacceptable. Multilevel linear modeling was performed with participant responsiveness and other implementation dimensions predictors. Intervention outcomes were intuitive eating and body esteem.

**Results:**

Unacceptable adaptations were significantly associated with providers’ self-efficacy (*r_s_*(23) = .59, *p* = .003) and past experience with facilitating the intervention (*r*(23) = .47, *p* = .03). Participant responsiveness showed a significant interaction between time and home practice completion (*B* = .07, *p* < .05) on intuitive eating scores.

**Conclusion:**

Except for participant responsiveness, other implementation dimensions did not moderate outcomes. Implications for future research and practice are discussed.

Health care professionals and researchers call for a need to intervene more effectively to reduce the disease burden associated with high body mass index (BMI) (GBD, 2015 Obesity Collaborators, 2017; Ng et al., 2014). Long-term healthy lifestyle changes seem to be a common ground for many health care professionals, although many different approaches are proposed to achieve this goal. Among the approaches focusing on health behaviors, the Health At Every Size (HAES®) movement is one of the most referenced (Cadena-Schlam & López-Guimerà, 2015). It advocates for health gains without necessarily losing weight and promotes intuitive eating, active lifestyle and self-acceptance. It also aims to stop weight-related stigma (Burgard, 2009). HAES® interventions accumulate more and more empirical evidence of their efficacy on health-related outcomes (Ulian et al., 2015), as well as psychological well-being and eating behaviors (Clifford et al., 2015). This approach has been suggested as a promising new direction in the public health sphere for long lasting behavioral changes (Bombak, 2014), although most research so far has been conducted in well-controlled settings. In this regard, Penney and Kirk (2015) pointed out the need for empirical studies to be conducted in a wider range of population to move the ‘reframing obesity debate’ forward. Studies in real-world settings allow not only to test evidence-based interventions within a more representative sample of a targeted population, but also to take into consideration many important yet overlooked factors, such as program implementation integrity, sociopolitical context, funding, and organizational characteristics. These factors can contribute to greater heterogeneity in responses but are unfortunately underreported in studies despite being known as crucial (Allen et al., 2012; Glasgow et al., 2012; Peters et al., 2014). Yet this knowledge gap is expected as translating evidence-based interventions into real-world settings can be challenging, especially in health and social care science (Hasson, 2010).

Surprising outcomes can result from implementing an intervention in a natural environment (Domitrovich & Greenberg, 2000; Durlak & DuPre, 2008). The assessment of implementation is therefore useful to interpret outcomes accurately, namely to determine whether they are attributable to the intervention’s theoretical components or the integrity of its application (Durlak & DuPre, 2008; Helmond et al., 2012; Mowbray et al., 2003). Most importantly, this can provide a possible explanation when observing weaker (or an absence of) results (Dobson, 1980). Implementation has been widely reported as influencing intervention outcomes, the magnitude of mean effect sizes being two to three times higher when studies monitor implementation in comparison with studies that do not (Durlak & DuPre, 2008). Implementation monitoring indeed unveils the highest potential benefits of an intervention (Cutbush et al., 2017; Elliott & Mihalic, 2004) by providing support to program instigators to increase the quality of delivery of the intervention. It also leads to a better understanding of the setting factors that foster or hinder outcomes, the processes by which they operate and how they can be improved (Carroll et al., 2007; Dobson, 1980).

Program implementation has been conceptualized in many ways and lacks in standardization regarding its nomenclature (Toomey et al., 2020). One of the most comprehensive conceptualizations encountered in the literature stems from Durlak and DuPre (2008), where they have envisioned implementation as a multidimensional construct grouping several aspects identified as: (1) adherence (fidelity is often used interchangeably), which refers to the degree to which an intervention is delivered as intended; (2) dosage, the *quantity* of the program actually delivered; (3) quality of delivery, referring to the skills with which the intervention was provided (e.g. clarity of instructions, ability to interact with participants); (4) participant responsiveness, the degree to which a participant displays interest in the intervention; (5) program differentiation, meaning the uniqueness of the program in comparison with other interventions; (6) monitoring of the control/comparison conditions; (7) program reach, meaning the scope of the program, and finally; (8) adaptation, referring to the changes that were made to the original program while it has been delivered. Based on this theoretical frame, Berkel et al. (2011) provided evidence that all these dimensions have been positively associated with program outcomes.

The Durlak and DuPre’s (2008) model was used in the current study because adherence (fidelity) is specifically distinguished from adaptations, which have been traditionally seen as a lack of fidelity (Blakely et al., 1987). The degree to which interventions are expected to be implemented with fidelity vary greatly from one perspective to another (Cutbush et al., 2017), and can be debated. Supporters of strict adherence are indeed opposed to those who adopt a more flexible view of fidelity, where adaptations are ‘allowed’ if they do not compromise the intervention core components (Cohen et al., 2008). Core components are defined as ‘the most essential and indispensable components of an intervention practice or program’ and are thought to determine the success of the intervention (Gould et al., 2014). Adaptation supporters argue that they could preserve and even enhance program effectiveness by making it more relevant to a set of diverse audiences and culturally competent (Castro et al., 2004). Interestingly, adaptations have both been associated with better and worse program outcomes (Stirman et al., 2013). This inconsistent body of literature is leading researchers to think that some modifications could indicate decreases in fidelity, while others embrace the intended purpose of the intervention. Yet treatment manuals cannot possibly list exhaustively in advance what behaviors and adaptations are acceptable or proscribed. Stirman et al. (2013) have thus highlighted the relevance of determining empirically core components of an intervention and coding adaptations in addition to fidelity monitoring.

Few studies include more than two components of implementation (Durlak & DuPre, 2008). Documenting the effects of several facets of implementation integrity on program outcomes at once is however relevant to better understand which dimensions account for the most variance (Giannotta et al., 2019). Within our field of research, some weight management programs have been implemented in a real-world setting and have addressed the issue of implementation (Campbell-Scherer et al., 2014; Damschroder & Lowery, 2013; Lombard et al., 2014). However, those studies used either different conceptualization frameworks that did not focus on individual-level outcomes, or they did not explicitly report outcomes in conjunction with a comprehensive assessment of implementation integrity. In the specific case of the HAES® approach, our research group would be, to our knowledge, the first to investigate the effects of implementation on program outcomes.

The purpose of this study is to explore to what extent does implementation integrity moderate outcomes of a disseminated HAES® intervention within the community. This study is in line with previous publications reporting on its program effectiveness (Bégin et al., 2018; Carbonneau et al., 2016), and herein focuses on the effects of implementation. More particularly, it examines implementation dimensions standing at two different levels: program participants (participant responsiveness) and providers (dosage, adherence, adaptation). It should be noted that adaptations were classified as either acceptable or unacceptable by instigators (according to the core components of the program). We hypothesized significant positive associations between all dimensions of implementation, with the exception of unacceptable adaptations that were assumed to be detrimental and negatively correlated with the other dimensions. We also hypothesized program outcomes to vary significantly across sites of implementation and to be predicted by implementation dimensions.

## Method and materials

### Intervention

‘*Choisir de maigrir?*’ (CdM?) (What about losing weight?) is a HAES®-based intervention for women which promotes intuitive eating and self-acceptance, following the example of the fat acceptance and size diversity movement. The intervention consists of 13 weekly three-hour sessions, plus an intensive six-hour day, provided in small groups of 10–15 participants and led by a social worker or psychologist as well as a dietitian. It aims to develop healthy ways of coping with weight management such as reevaluating eating habits and food intake, enjoying physical activity and being critical towards diets. By the end of the program, it prompts participants into free and informed decision-making about losing weight. A realistic action plan of behavior changes customized to their own personal situation is then designed accordingly. As such, the success of intervention of CdM? relies on outcomes reflecting a healthier relationship with oneself such as improvement of body esteem and intuitive eating (Bégin et al., 2018; Carbonneau et al., 2016). CdM? has the special feature of giving participants the opportunity to lead some discussions and to customize their goals and actions to achieve them, in a way to encourage empowerment throughout the program. Main themes addressed during CdM? program with examples of activities has been previously published (Carbonneau et al., 2016). The efficacy of CdM? has been assessed several times since then and revealed mostly positive outcomes on eating behaviors and psychological variables (Gagnon-Girouard et al., 2010; Mongeau, 2004; Provencher et al., 2007; Provencher et al., 2009).

### Cdm? Dissemination overview

The current research took place in the context of a massive dissemination of CdM? in Health and Social Services Centres (HSSC) across the province of Québec in response to the public health action plan led by the Ministère de la santé et des services sociaux (MSSS, Ministry of Health and Social Services) to promote healthy habits and prevent weight-related issues (MSSS, 2012). The dissemination of CdM? was entrusted to ‘ÉquiLibre’, a Québec-based nonprofit organization aiming at preventing and reducing issues related to weight and body image in the population (Groupe d’action sur le poids ÉquiLibre, 2021). They ensured the training of all providers with a free five-day seminar. They also provided them with a turnkey intervention toolkit including a detailed step-by-step description of the intervention, as well as an explanation of the theoretical rationale behind the program, a comprehensive review of literature on weight management, intervention materials, practical advice for starting the program and videoclips from previous CdM? facilitation (Groupe d’action sur le poids ÉquiLibre, 2005).

### Procedure

HSSCs from across the province of Quebec (Canada), spread over 9 different regions from Quebec (80% urban and 20% rural areas), were provided with the instructions and assessment materials by the research team. They were entirely in charge of recruitment and data collection, and delivery of the intervention. Participants of CdM? were recruited starting from September 2010 to December 2011. Procedures were approved by the research ethics committee (REC) of the Health and Social Services Agency of Montreal. They were also ratified by each HSSC local ethics committee. This study was conducted following the principles of the Declaration of Helsinki.

CdM? providers gave participants a series of pen-and-paper questionnaires to complete at home at baseline (T1 = 0 month), after the intervention (T2 = 4 months) and at 1-year follow-up (T3 = 16 months). Only data from T1 to T2 were used as this study focuses only on the processes related to the implementation. Participants also had to complete a sociodemographic questionnaire at baseline, and a feedback survey about CdM? at the end of the intervention (T2). Providers completed an evaluation grid which was used as a reminder at the end of every session to report the conduct of each planned activity. These reminders were all sent back to the research team by mail at the end of the intervention. Providers had to report in their grid the extent in which the activity was performed with integrity, meaning whether the activity was: (1) performed accordingly to the manual; (2) performed with some modifications; or (3) not performed. They also had to report the length of each activity and, if required, described the modifications they made. An individual semi-structured interview was then conducted by phone at the end of the program with each provider in order to better understand the course of implementation in their respective HSSC. The interview was recorded and lasted approximately one hour. The research goals as well as the independency of sources of funding were recalled at the beginning of each interview. The interview guide had 30 questions that were derived from evidence-based factors known to influence implementation integrity (Durlak & DuPre, 2008), and in relation with the core components identified by the instigators of the program (see *Identification of CdM? core components below*). Qualitative data from the reminder and interview verbatim transcripts were imported in NVivo v.9.0 for analysis.

#### Identification of CdM? core components

**Figure 1.**
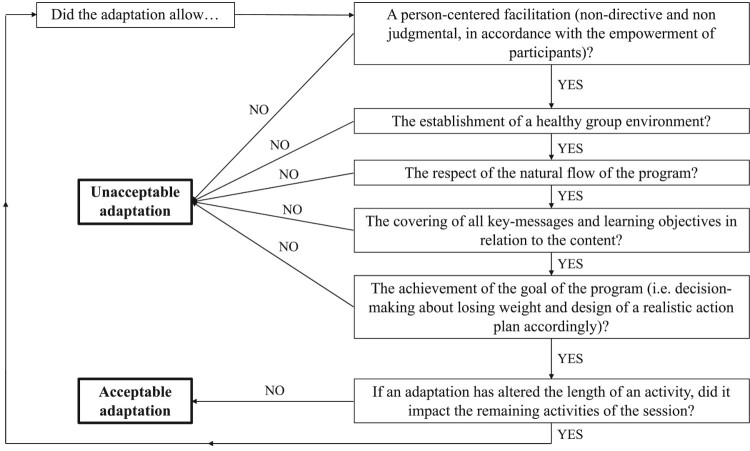
Decisional algorithm about the acceptability of adaptations made to CdM?.

The initial instigators of CdM? (program developers) were invited to provide the research team with their subjective insight on core components of the program. Instigators had first identified primary theoretical core components of the program, namely: the non-diet, self-acceptance and empowerment approaches. They completed a grid listing each activity of the program, which were rated according to their degree of importance (1 = very important, 2 = somewhat important and 3 = slightly important) and then associated with core components. They were also asked to comment on the level of flexibility they would allow adaptations on (a) the holding of the activity, (b) its content, (c) its facilitation style and (d) its length. Grids were sent back to the research team and led to the creation of a decisional algorithm allowing the classification of modifications to the program made by providers into two distinct categories: (1) acceptable adaptations and (2) unacceptable adaptations (see Figure 1). Five main core components resulted from the several qualitative analyses (Samson, 2015): (1) empowerment; (2) healthy group environment; (3) natural flow; (4) learning objectives (key-messages regarding the non-diet and self-acceptance approaches); (5) aim of the program (informed decision-making process toward the design of a personalized action plan).

### Sampling

#### Participants

216 adult women from the community participated in CdM?, nested within HSSCs across the province of Quebec (Canada). The intervention was opened to any woman seeking treatment for eating or weight-related problems. Apart from being aged 18 years-old and over, no formal exclusion criteria were used for restricting entrance to the program. A mandatory information session was held prior to the program to ensure their understanding of the themes covered during the program. Participants reporting a pregnancy over the course of the study (n = 5) were excluded from the analyses for sample homogeneity reasons. 162 participants completed the questionnaire at the end of the intervention and were used for the analyses.

#### Providers

**Figure 2.**
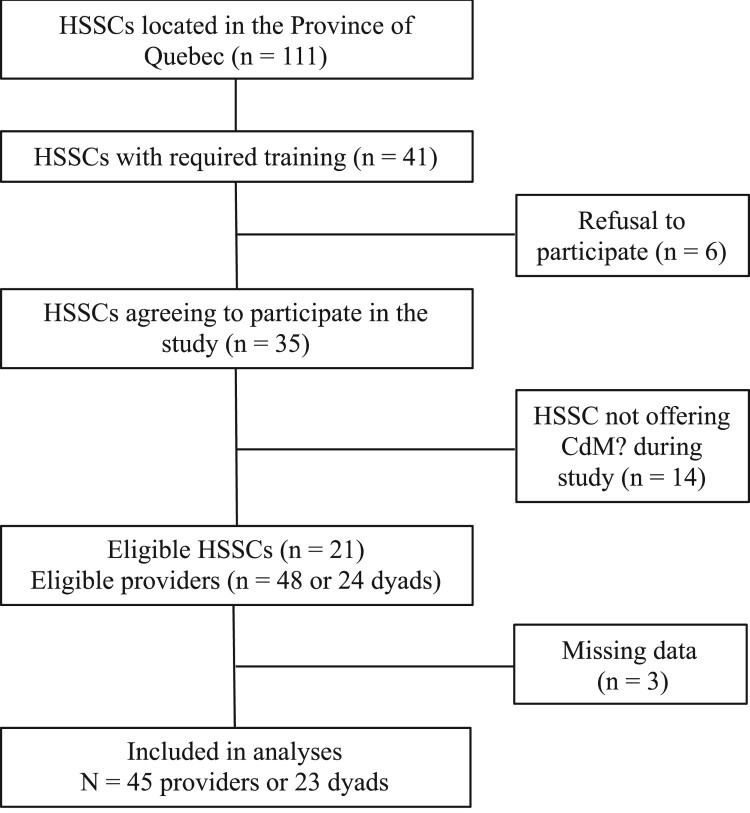
Flowchart of providers recruitment.

Recruited dyads of providers consisted of a registered dietitian and a psychologist or social worker who were already trained by ÉquiLibre and engaged into delivering CdM?. All HSSCs that had at their disposal a trained dyad of CdM? providers were approached for recruitment (n = 41). From these invitations, 6 refused to participate in the study and 14 HSSCs were not giving the intervention at the time of data collection, thus resulting in 21 eligible HSSCs and a total of 24 dyads (since some HSSCs have trained several dyads of providers across different establishments). All providers agreed to participate in the study, although three of them did not complete data collection, one for medical reasons and the remaining two, due to lack of time. The final sample size of providers was n = 45 (see Figure 2 for flowchart of HSSCs/providers recruitment).

### Measurements

#### Implementation dimensions.

##### Adherence

Adherence was computed by listing all activities that were completed accordingly to the program. This number was then divided by the total number of activities (n = 124) and multiplied by 100 to obtain a percentage of adherence to the curriculum. Adherence has been documented in the past as a percentage of program activities completed (Dusenbury et al., 2005). As mentioned earlier, measure of adherence results from providers’ evaluation grids that were completed at the end of each session (see procedure).

##### Dosage

The amount of exposure to CdM? was calculated from the length of activities reported by providers in their reminder grid. For each HSSC, the dosage was computed from dividing the amount of time of program actually delivered by the total alleged duration of the program, then multiplied by 100 to obtain a percentage of exposure.

##### Acceptable adaptation

Adaptation is the number of modifications made to the program curriculum per providers’ dyad that were classified ultimately as acceptable adaptations according to the standards of instigators. Adaptations could either refer to modifications or additions to the program, as long as they were not altering core components of CdM?. Examples of acceptable adaptations would be adding an optional activity planned in the manual, providing additional explanations, examples or visual support: ‘an example […] of these documents (activity journal and compilation of activities) was photocopied and given to participants as examples to make their own compilations at home’; or bringing together activities with similar themes: ‘Moved the [presentation on the body's resistance to weight loss] to Session 12 (I become critical of diets)’.

##### Unacceptable adaptation

This variable herein refers to the number of modifications performed per providers’ dyad (e.g. additions, removals, changes in the progression of activities, alteration of core components) that were classified as unacceptable adaptations by the decisional algorithm. They could be, for instance, the addition of a new activity without consulting participants, ‘We’ll do the taboo foods exercise (which is not planned in the program) […]’; while omitting theoretical content, ‘[The physiological consequences of obesity of the energy balance presentation sheet] not completed […]’, or limiting group discussions and interventions, which was considered to obstruct empowerment.: ‘[…]we asked women to limit their interventions when giving feedback about their visualization exercise’. It is to be noted that all removal modifications were classified as unacceptable.

##### Participant responsiveness

A questionnaire developed by the research team was used to assess participants’ responsiveness to CdM?. We hereby define participant responsiveness as a multidimensional construct referring to involvement and interest to the program that includes attendance, subjective improved knowledge, home practice completion and satisfaction. We also included goal achievement as another evidence of their responsiveness to the program. Qualitative information was collected in concomitance to the quantitative assessment of their responsiveness, using written feedback on the intervention. For instance, participants were asked to provide explanations as to why they would not have met their goals, or reasons behind their assessment of their satisfaction towards each activity.

*Goal achievement.* Participants were asked whether they have achieved their main goal over the course of the program, to which they could either answer (1) yes, (2) more or less or (3) no.

*Attendance.* The attendance of participants to each session of CdM? was documented by providers of the program who reported it at the beginning of each session. Attendance was computed by summing up attendance to each session by the end of the program.

*Subjective Improved Knowledge (SIK)*. Participants were assessed on their improvement of knowledge on theoretical subjects thoroughly discussed during the program (e.g. energy balance, determinants of weight regulation, physical activity, body dissatisfaction, weight-loss products and programs). Participants could answer either ‘1 = slightly’, ‘2 = moderately’ or ‘3 = a lot’. A mean score was calculated by averaging scores for the six learning components. Higher scores indicate higher improved knowledge. Internal consistency was acceptable (α =  .70).

*Home Practice Completion (HPC).* Participants self-reported on a Likert-scale how often they put into practice the several methods and problem-solving exercises learned during the program (1 = never; 5 = very often). Ten behaviors were assessed: (1) be aware of false hunger; (2) do an enjoyable substitutive activity not related to food; (3) listen to your body; (4) taste the food you eat; (5) feel and respect satiety signals; (6) choose the desired foods; (7) relax; (8) be active; (9) express your feelings; and (10) assess difficult situations. A mean score was then computed, where higher scores reflect higher home practice completion. Internal consistency was acceptable (α =  .73).

*Satisfaction.* Participants were asked to rate their level of satisfaction towards the several types of activities conducted during the program. They were asked: ‘Are you satisfied with the following activities?’, to which they could answer ‘1 = slightly’, ‘2 = moderately’ or ‘3 = a lot’. More specifically, nine types of activities were assessed: (1) energy balance assessment; (2) true signals of hunger exercise; (3) tasting exercise; (4) role-playing; (5) modeling dough exercise; (6) theoretical/conceptual presentation; (7) visualization exercises; (8) relaxation/mindfulness exercise; and (9) action plan design. A final score was averaged from all types of activities. Higher scores indicate higher satisfaction. The internal consistency for this variable was however lower than the other measurements of responsiveness (α = .61). This weaker reliability may be attributed to the eclectic and multidimensional nature of the program, which relies on significantly different types of activities.

#### Provider characteristics

##### Experience

Providers were asked to report the number of years of experience they had in their respective professional area (dietitian or social worker/psychologist). A mean score was computed for each providers’ dyad.

##### Cdm? Experience

CdM? experience refers to the specific program-related experience of the providers, meaning how often they have offered CdM? in the past in their current HSSC or other establishments.

##### Self-efficacy

Providers were assessed on three theoretical founding pillars of CdM?: (1) the non-diet approach; (2) the self-acceptance approach and (3) the empowerment approach. Providers were asked: ‘To which degree do you feel able to convey information about [e.g. the non-diet approach]?’ and had to report it on a 5-point Likert-scale (1 = not at all, and 5 = entirely). They were similarly assessed about a set of relevant skills for multipatient interventions: (1) group dynamic facilitation, (2) handling emotional participants, (3) handling quiet participants, (4) handling overwhelming participants, (5) managing auto-facilitated sessions, (6) managing dyadic facilitation, and (7) adopting a non-directive style of facilitation. A mean score averaging the up-mentioned items was computed for each provider and dyad of providers, resulting in a mean self-efficacy score. Higher scores indicate higher sense of self-efficacy. Internal consistency was good (α = .83).

#### Program outcomes

##### Intuitive eating

The Intuitive Eating Scale (IES) is a scale of 21 items displaying a total score as well as 3 subscales: (1) Eating for physical rather than emotional reasons, (2) Unconditional permission to eat when hungry and what food is desired, and (3) Reliance on internal hunger and satiety cues (Tylka, 2006). This questionnaire informs globally to which point an individual is inclined to eat accordingly to his hunger and satiety signals, as well as being able to listen to its body in order to guide what, when and how much to eat. For the purposes of this study, the total score was used as a main outcome rather than examining each subscale individually. Many studies have supported the construct validity of this scale with women (Tylka & Van Diest, 2013). The Cronbach alpha coefficient for the total score was above .70 at baseline and post-intervention (α = .78 and .82 respectively). It indicated good internal reliability in the current study.

##### Body esteem

The Body Esteem Scale is a validated 23-item questionnaire composed of 3 subscales, namely the (1) Appearance, (2) Weight and (3) Attribution subscales (Mendelson et al., 2001). While the first refers to general self-appreciation about appearance, the second focuses more on weight satisfaction strictly speaking. The attribution subscale refers to social attributions made about one’s body and weight. The BES has been validated in adults of a wide range and provided good test-retest reliability, as well as convergent and discriminant validity (Mendelson et al., 2001). Only the appearance subscale was used to assess the construct of body esteem (hereafter BESAP). This subscale has 10 items for which a mean is computed. Items range from 0 to 4 on a Likert scale (0 = never; 4 = always), lower scores indicating lower body esteem. The choice of this subscale relied mostly on philosophical considerations, appearance being conceptually closer to what is addressed in CdM? sessions. Internal consistency for this scale was good (respectively α = .89 at baseline and α = .90 at post-intervention).

### Statistical methods

Using SPSS v.25, we performed descriptive statistics and bivariate pairwise Pearson correlations between variables, while we used Spearman correlations (r_s_) when assumptions could not be met for parametric statistic tests (Aggarwal & Ranganathan, 2016). Univariate outliers identified with the outlier labeling rule sustained a 90% winsorization, though unwinsorized data is shown in descriptive statistics. Independent-samples t-tests or Mann–Whitney *U* tests were also performed to compare providers by their occupation. A two-tailed *p*-value of .05 was employed as the criterion of statistical significance for all tests. Bonferonni correction was used when conducting multiple testing. A three-level multilevel (hierarchical) linear modeling (MLM) (time (1), and participants (2) nested in HSSC implementation sites (3)) was performed through SPSS MIXED MODELS for the examination of the effect of implementation dimensions on prediction of outcomes of the intervention over time. MLM is an appropriate statistical method when data is nested in units of a higher level of analysis by allowing the study of the relationship between a dependent variable and one or more explanatory variables without violating assumptions of independence in linear multiple regression. Assumptions regarding normality of residuals and absence of outliers using Mahalanobis distance were assessed for the set of predictors. A null model in which no covariates were added (intercepts-only model) was tested for each outcome to provide intraclass correlation among each level of the hierarchy. Models were then tested with centered predictors (implementation dimensions, participant responsiveness and time).

## Results

Descriptive statistics of CdM? participants are presented in Table 1. Descriptive statistics of CdM? providers are presented in Table 2. On average, providers had 15.75 years of experience and facilitated the CdM? program 3.76 times in the past (see Table 3 for means and standard deviations per dyad). No significant differences were found between providers accordingly to their occupation, except for self-efficacy in handling emotional participants. The Mann–Whitney-U test indicated that psychosocial professionals (*M* = 4.64) reported greater self-efficacy on this skill than dietitians (*M* = 3.68), *U *= 111.5, *p *= .001, ^2 ^= .25.

**Table 1.** Descriptive statistics of CdM? participants at baseline.

Demographic variables	*n*	min	Max	*M*	*SD*
Age	162	21.00	77.00	51.38	10.56
BMI	123	23.95	64.55	36.55	6.91
Race (%)					
Caucasian	156			96.90	
Black	1			.60	
Latino	4			2.50	
Family Income (CA$) (%)					
<39,000	71			48.00	
40,000–79,000	40			27.00	
80,000 and above	37			25.00	
Employment (%)					
Student	1			.60	
Employed	93			57.70	
Unemployed/retired	60			37.30	
Other	7			4.30	
Education (%)					
Elementary School	3			1.90	
High School	56			35.00	
College	49			30.60	
University	52			32.50	
Living area (%)					
Rural	36			22.20	
Urban	93			57.40	
Suburban	33			20.40	
Program Outcomes					
Intuitive Eating (IES)	162	1.71	4.24	2.78	.51
Body Esteem (BESAP)	162	0.00	3.60	1.18	.73

**Table 2.** Descriptive statistics of CdM? providers’ characteristics by their occupation.

Variables	Dietitian					Psychosocial professional						
	*N*	*min*	*max*	*M*	*SD*	*n*	*Min*	*max*	*M*	*SD*	*t*/*U*	*p*
Experience	22	3.00	27.00	14.05	7.82	23	3.00	35.00	16.98	8.26	−1.22	.23
CdM? Experience	22	1.00	22.00	4.09	4.63	23	1.00	9.00	3.57	2.33	−.09	.93
Self-efficacy												
Empowerment approach^a^	14	3.00	5.00	4.43	.65	16	3.00	5.00	4.75	0.58	79.00	.10
Self-acceptance approach^a^	11	4.00	5.00	4.55	.52	15	4.00	5.00	4.87	0.35	56.00	.07
Non-diet approach^a^	16	2.00	5.00	4.69	.79	17	3.00	5.00	4.53	0.72	114.50	.32
Group Facilitation^a^	22	3.00	5.00	4.68	.65	22	3.00	5.00	4.43	0.76	198.00	.21
Handling emotional participants^a^	22	2.00	5.00	3.68	1.01	22	3.00	5.00	4.64	0.58	111.50*	.001
Handling quiet participants	22	2.00	5.00	4.11	.90	22	3.00	5.00	4.39	0.75	−1.09	.28
Handling overwhelming participants	20	2.00	5.00	3.55	.95	18	3.00	5.00	4.28	0.9	107.00	.02
Managing auto-facilitated sessions	22	2.00	5.00	4.27	.88	20	3.00	5.00	4.25	0.79	.09	.93
Managing dyadic facilitation^a^	22	4.00	5.00	4.80	.40	19	2.00	5.00	4.68	0.75	208.50	.99
Adopting a non-directive style of facilitation	22	3.00	5.00	4.09	.68	22	3.00	5.00	4.36	0.73	−1.28	.21

^a^ Wilcoxon-Mann-Whitney test performed.

*Indicates significance of the test, where α = .004 was used with Bonferonni correction (.05 /12).

**Table 3.** Descriptive statistics and correlations between participant responsiveness, providers’ characteristics, other implementation dimensions.

Variables	*n*	min	max	*M*	*SD*	1.	2.	3.	4.	5.	6.	7.	8.	9.	10.	11.
**Participant responsiveness**																
Goal achievement (%)																
Yes	108			67.90												
More or less	39			24.50												
No	12			7.50												
1. Attendance	150	10.00	14.00	13.17	1.02	–										
2. SIK	161	1.33	3.00	2.60	.37	−.05	-									
3. HPC	160	2.00	4.90	3.61	.44	−.08	.09	-								
4. Satisfaction	158	1.88	3.00	2.74	.26	−.04	.37**	.26**	-							
**Provider characteristics**																
5. Experience (years)	23	4.50	25.00	15.75	6.02	-.06	.08	.17	–							
6. CdM? experience	23	1.00	15.00	3.76	3.09	.07	.01	−.06	.19	–						
7. Self-efficacy	23	3.95	4.97	4.39	.27	−.05	.15	.03	.26	.42*	–					
**Implementation dimensions**																
8. Dosage (%)	23	70.07	102.81	90.28	8.80	.00	−.11	.10	.15	−.07	−.22	.06	–			
9. Adherence (%)	23	46.77	95.97	75.77	13.17	−.15	.25*	−.07	.10	−.04	−.27	−.08	−.08	–		
10. Acceptable adaptation	23	3.00	27.00	10.00	5.49	−.01	−.15	.15	.04	−.10	.06	.23	.16	−.43	–	
11. Unacceptable adaptation	23	2.00	18.00	7.34	3.52	−.09	−.09	.11	−.08	−.12	.47*	.59**	−.17	−.32	.30	–

*p < .05; **p < .01*.*

Means and correlations regarding participant responsiveness and other implementation dimensions are shown in Table 3. Bivariate correlations between subdimensions of participant responsiveness revealed positive associations between subjective improved knowledge and satisfaction (*r_s_* = .37, *p* < .001), and home practice completion and satisfaction (*r_s_* = .26, *p* = .001), both with a medium effect size. Unacceptable adaptations showed strong positive associations with providers’ dyads CdM? experience (*r* = .47, *p* = .03) and their overall self-efficacy (*r_s_*_ _= .59, *p* = .003), while self-efficacy correlated with CdM? experience (*r_s_* = .42, *p* < .05). Dosage did not correlate significantly with any other variable. Adherence correlated positively with subjective improved knowledge (*r_s_* = .25, *p* = .01), but with no other variable.

The null model tested with intercepts only resulted in a total mean score on IES of 2.93 (*t* (18.91) = 50.28, *p* < .001), with $\sigma_{error\;}^{2}\;=\;$.22, *p* < .001, $\sigma_{participant\;}^{2\;}\;$  .07, *p*  = .03, and $\sigma_{HSSC}^{2}$ = .03, *p *= .11. Intraclass correlations (ICC) were calculated accordingly, $\rho_{1}$_ = _.68, $\rho_{2}$_ = _.21 and $\rho_{3}$_ = _.10 respectively for intraindividual residual error, participant and HSSC levels. We similarly obtained a total mean score on BESAP of 1.39 (*t* (15.20) = 20.72, *p *< .001), with $\sigma_{error}^{2}$_ _= .21, *p* < .001, $\sigma_{participant}^{2}$  .44, *p* < .001, and $\sigma_{HSSC}^{2}$_ _= .004, *p* = .89. ICC were then calculated, resulting in $\rho_{4\;}\;$  .33, $\rho_{5\;}\;$  .67 and $\rho_{6\;}\;$  .01. As no support was found to perform 3-level modeling, we examined predictors related to implementation dimensions using two-level models. It is to be noted that only the intercept and residual deviations were entered as random effects, since slope and slope*intercept covariance deviations did not allow the models to converge properly. As such, time was entered as a fixed effect only and an identity variance-covariance matrix was used. The models were significantly better fitted to the data than the ones with intercepts only, χ^2^ (11, N = 162) = 636.03–416.78 = 219.25, *p < *.001 for IES and χ^2^ (11, N = 162) = 259.03, *p < *.001 for BESAP. Results of models are presented in Tables 4 and 5 with participant responsiveness predictors only, as the other implementation dimensions did not interact with time neither for intuitive eating nor for BESAP (data available in supplemental material). For intuitive eating, participants on average had an IES score of 2.71 at baseline (*p* < .001), which significantly deviated between participants, and had an average increase in IES of .08 per month over the course of the intervention. Home practice completion was the only significant predictor interacting with time (${\text{β}}_{X2\;time}$_ _= .07, *t*(133.52) = 2.13, *p* < .05), showing an additional increase in scores of IES of .07 per month for each increase of one unit of home practice completion. Other predictors did not have a significant effect on the slope. For body esteem, Table 5 displays significant interindividual differences around intercepts, which was 1.02 on average, as well as a significant slope of .08 (*p* < .001). Participants had a lower intercept of .46 points for each increase of one unit for subjective improved knowledge, meaning that those who reported higher scores on subjective improved knowledge had a lower body esteem at baseline. No other significant effect was found among participant responsiveness predictors.

**Table 4.** Multilevel analysis of intuitive eating score by time and participants’ responsiveness.

Fixed effects	Coefficient	SE	df	*t*
Intercept	2.71***	.08	225.78	34.12
Attendance (X_1_)	.05	.04	225.78	1.15
SIK (X_2_)	−.07	.13	225.78	−.57
HPC (X_3_)	.13	.10	225.78	1.23
Satisfaction (X_4_)	.04	.19	225.78	0.21
Objective Achievement (X_5_)				
Yes	.09	.10	225.78	.93
No	.00			
*Level 1: intraindividual effects on intuitive eating*				
Time	.08**	.02	126.83	3.51
*Level 2: between participants effects on intuitive eating*				
Attendance (X_1_)*time	−.001	.01	129.65	−.10
SIK (X_2_)*time	−.02	.04	126.19	−.63
HPC (X_3_)*time	.07*	.03	133.52	2.13
Satisfaction (X_4_)*time	−.003	.06	131.67	−.05
Objective Achievement (X_5_)*time				
Yes	.04	.03	127.83	1.32
No	.00			
**Random effects**	**Variance**	**SE**		**Wald-Z**
Residual deviation	.16***	.02		7.74
Intercept deviation	.10***	.02		3.88

*p < .05; **p < .01.; ***p < .001.

**Table 5.** Multilevel analysis of body esteem (appearance subscale) score by time and participants’ responsiveness.

Fixed effects	Coefficient	SE	df	*T*
Intercept	1.02***	.11	177.51	9.05
Attendance (X_1_)	.02	.06	177.51	.41
SIK (X_2_)	−.46*	.18	177.51	−2.54
HPC (X_3_)	.18	.15	177.51	1.23
Satisfaction (X_4_)	.19	.27	177.51	.72
Objective Achievement (X_5_)				
Yes	.19	.14	177.51	1.41
No	.00			
*Level 1: intraindividual effects on body esteem*				
Time	.08***	.02	122.34	3.61
*Level 2: between participants effects on body esteem*				
Attendance (X_1_)*time	−.02	.01	123.83	−1.84
SIK (X_2_)*time	.01	.04	122.00	.20
HPC (X_3_)*time	.04	.03	125.82	1.20
Satisfaction (X_4_)*time	−.04	.06	124.88	−.70
Objective Achievement (X_5_)*time				
Yes	.03	.03	122.87	1.19
No	.00			
**Random effects**	**Variance**	**SE**		**Wald-Z**
Residual deviation	.15***	.02		7.69
Intercept deviation	.36***	.06		6.59

*p < .05; **p < .01.; ***p < .001.

## Discussion

This study aimed to examine the effect of implementation on outcomes of a community-based HAES® intervention. Regarding our main research goal, our preliminary analysis did not support evidence of significant variability across implementation sites and implementation dimensions did not moderate outcomes, except for home practice completion. While these results go against the main body of literature stating that implementation integrity moderates program outcomes, we call for cautiousness in their interpretation for several reasons. First, it should be reiterated that ‘no evidence of effect’ is not to be confounded with an ‘evidence of no effect’ (Ranganathan et al., 2015), and we might have encountered a type II error, perhaps due to the limitations regarding the measurements used or the sample size. Secondly, it is possible that CdM? has been given sufficiently faithfully by providers in a way to reach a ‘good-enough’ threshold allowing participants to benefit from the program at scale. In the same vein, it could mean that the adaptations performed, regardless of whether they were classified as acceptable or unacceptable by the algorithm, were either in line with the intended philosophy of the intervention or inconsequential. This would be possible given that the participant responsiveness was globally positive (quantitatively and qualitatively). Thirdly, we must point out that CdM? is a program that is also facilitated by participants, and that group dynamics take up a lot of space in the program. This could partially explain as to why no provider effect was found, as opposed to therapist effects usually accounting for around 7% of outcomes (Schiefele et al., 2017). Fourthly, the HAES® approach is known to have multiple outcomes and so, it is not because no effect of implementation was found on the two outcomes chosen in this study that it prevents it from influencing other outcomes, such as reducing maladaptive eating behaviors or other psychological well-being measures. Nevertheless, we should mention that other studies have found similar results than ours, where no effect was found for implementation dimensions except for participant responsiveness (Giannotta et al., 2019). This study is thus in line with a body of literature highlighting the importance of participant responsiveness (Berkel et al., 2011). A study has even recently found that participant responsiveness had a direct effect on outcomes, rather than being a mediational influence in the association between quality of delivery and outcomes (Doyle et al., 2018).

An interesting result emerging from our analysis was that home practice completion had a positive effect on change over time in intuitive eating. It is concordant with basic principles conjured in behavioral science, that emphasize the importance of performing successive approximations of the behavior to experience change (Jackson, 1997). Although this finding is rather self-explanatory, it emphasizes that intuitive eating improves over time through practice. Therapists who teach intuitive eating could therefore put emphasis on practicing at home the principles seen in session and present it as a skill that can be learnt. On the other hand, no significant participant responsiveness predictor was found for body esteem. It is possible that the choice of variables was less appropriate for this particular outcome. It might have been useful, retrospectively, to have assessed participant responsiveness in a way that would capture satisfaction with group exchanges, such as feeling accepted by the group and being treated in a non-judgmental manner. Perhaps the atmosphere of the group could have been used in predicting change in participants’ body image.

Our study also led us to take a closer look at the implementation process. Although modifications were expected, the extent to which certain dyads have not followed the program cursus was quite important. This should however be interpreted with caution while considering the self-reported nature of our measurement, which can deviate from the true course of events (as opposed to the use of observers rating providers’ behaviors). Meanwhile, results in terms of adherence are difficult to interpret since no benchmark was defined prior to the conduct of this study, again suggesting caution in results interpretation. We also noted that each providers’ dyad has performed acceptable and unacceptable modifications to the program. Moreover, providers reported a very high sense of self-efficacy on theoretical pillars of the HAES® movement, as well as on skills required to facilitate a program in a group setting, regardless of their occupation. An exception to this would be the handling of emotional patients, as dietitians reported feeling less confident on this matter. This could indicate the relevance of having providers with complementary fields of expertise.

The associations between dimensions of implementation adherence did not significantly correlate with hardly any variables, contrary to our expectations. Only subjective improved knowledge correlated moderately with adherence, which could indicate that participants received more educational material when providers closely followed the curriculum of the program. Another surprising result related to unacceptable adaptations, where a moderate association found between providers’ self-efficacy, CdM? experience and performing unacceptable adaptations. These results appear counterintuitive as self-efficacy was found to be associated with adherence (Campbell et al., 2013; Thierry et al., 2022). However, it is important to recontextualize our results within the current study as the decisional algorithm departing acceptable from unacceptable adaptations, based on the instigators view of the program, seems especially unforgiving to providers’ initiatives. Indeed, not only did the algorithm not allow alterations of the learning objective, but it also severely restricted the way by which the program was given (e.g. empowerment, group environment, natural flow). Therefore, many adaptations, such as ‘doing a lot of mirroring during and after group discussions […]’, were classified as unacceptable, while they were not altering, in our opinion, core components *per se*. As such, it is likely that the providers confident in their abilities felt more comfortable to perform adaptations of greater extent. This could instead reflect on a good mastery of the program. Providers could also have either overreported or underreported the adaptations made. This could provide an alternative explanation as to why unacceptable adaptations were associated with self-efficacy. Overreporting adaptations, a downside from using self-reported methods in fidelity of implementation (Allen et al., 2012), could simply reflect conscientiousness from some providers.

In terms of participants’ responsiveness, it seems that most of them expressed genuine enthusiasm and engagement towards CdM?. They indeed reported high satisfaction towards activities of the program. The self-reported scores matched qualitative data as well, which was very positive in general. More than two thirds of participants reported having achieved their main goal by attending to CdM?, and those who responded not having met their goal recognized, however, that their goals changed throughout the intervention. Several expressed having new ‘insights’ regarding their issues, such as ‘needing to address their mental health problems prior to (their weight)’, while others held onto their goal to lose weight and expressed being now ‘better equipped’ for it. When participants reported dissatisfaction from their participation to the program, they generally mentioned having felt ‘overwhelmed by the amount of homework to do’ or ‘for not having invested as much (efforts) as they would have wanted to’. Regarding their satisfaction towards each activity, most participants’ feedbacks were generally positive, except for the play dough activity, which generated polarized reactions. This high responsiveness among participants could, all in all, reflect a high-quality implementation.

This study presents several strengths that are worth mentioning. First, this is, to our knowledge, the first study in the field of HAES® to examine outcomes of a disseminated and community-based intervention through the lens of implementation science. We might as well point out that we based our study on a model of implementation as it is recommended by the current guidelines in implementation science (Toomey et al., 2020). This brought us to consider several dimensions of implementation rather than only measuring treatment adherence. We also have used a mixed methodology, combining qualitative and quantitative data, to have a better understanding of implementation processes, which, once again, has been numerously recommended in the literature (Peters et al., 2014; Toomey et al., 2020). Another strength was the consideration of adaptations independently from adherence, even more so that we separated unacceptable from acceptable adaptations using an assessment of the intervention core components. Indeed, the measurement of treatment adherence only would have failed to capture the occurring of certain adaptations. However, this study also includes some important limitations to mention. The biggest limitation relates to the self-reported methodology used for measuring adherence and adaptations made to the program, which could have compromised the quality of the data by social desirability, omissions, overreporting and underreporting. It would have been more accurate to have these concepts measured using independent and exterior observers that would rate behaviors of providers, as well as to determine a gold-standard in terms of fidelity (or benchmark) that would have allowed, for instance, a categorization of low-quality implementation and high-quality implementation. Another limitation yet in regard to adaptations is the determination of essential components, which has been done retrospectively to the implementation of CdM?, using only the point of view of its instigators. It is also important to note that measurements of participant responsiveness were derived from a questionnaire developed by the research team, which is suboptimal in terms of fidelity and validity. For instance, quantifiers of frequency for the home practice completion could have been more precise, and objective assessment of knowledge change could have been used. As such, our results should be interpreted very cautiously.

In conclusion, our study showed the complexity of implementing a multi-dimensional intervention in a community-based setting. Our main analysis failed to demonstrate an effect of HSSC-level implementation on outcomes. While disappointing in terms of findings, this ‘absence of results’ could be seen positively, as it is possible that participants improved their intuitive eating and body esteem independently from *how* the intervention was given. It challenges the attention given to treatment adherence. Meanwhile, we found that participant responsiveness (home practice completion) had a positive effect on intuitive eating. More studies in the field are needed to explore which components of implementation matter the most, especially given the complexity of implementing and scaling-up interventions. The question of whether a ‘lack of adherence’ could have the same detrimental effect as the wrongdoing of unacceptable modifications to the treatment would also deserve, in our opinion, further investigation. More particularly, researchers should explore the impacts of different types of adaptations and better understand the context in which they are performed. In that regard, our study showed unexpectedly that unacceptable adaptations made to the program were associated with greater self-efficacy and experience with the program. Finally, more attention should be given to the monitoring of implementation in the field of obesity as it would step up the quality and accuracy of findings in future effectiveness studies.

## Acknowledgments

Many thanks to Hans Ivers, Ph.D., for his teaching of statistical analysis.

## Supplementary Material

Supplemental Material
