# Supplementary material for: Health At Every Size intervention® under real-world conditions: the rights and wrongs of program implementation
Source: Health Psychol Behav Med. 2022 Sep 30;10(1):935–55. doi: 10.1080/21642850.2022.2128357 (PMC9543046; doi:10.1080/21642850.2022.2128357)
Supplement: Supplemental Material [file RHPB_A_2128357_SM6709.docx]

$\eta$ $\sigma2error =$ $\sigma2participant$ $\sigma2HSSC$ $\rho1$ $\rho2$ $\rho3$ $\sigma2error$ $\sigma2participant$ $\sigma2HSSC$ $\rho4$ $\rho5$ $\rho6$ $\chi$ $\chi$ $\beta X2\cdot time$

**SUPPLEMENTAL MATERIAL**

Table 6. Multilevel analysis of intuitive eating score by time and other implementation dimensions

| **Fixed effects** |  | **Coefficient** | **SE** | **df** | ***t*** |
| --- | --- | --- | --- | --- | --- |
| Intercept |  | 2.80*** | .05 | 195.69 | 61.53 |
| Adherence |  | .01 | .01 | 195.69 | 1.29 |
| Dosage |  | .01 | .01 | 195.69 | 1.40 |
| Acceptable adaptation |  | .02 | .01 | 195.69 | 1.65 |
| Unacceptable adaptation |  | .05* | .02 | 195.69 | 2.34 |
| *Level 1: intraindividual effects on intuitive eating* | |  |  |  |  |
| Time |  | .08** | .01 | 107.84 | 5.91 |
| *Level 2: between participants effects on intuitive eating* | |  |  |  |  |
| Adherence*time |  | .00 | .00 | 108.95 | .37 |
| Dosage*time |  | .00 | .00 | 115.28 | .20 |
| Acceptable adaptation*time |  | .00 | .00 | 109.73 | .27 |
| Unacceptable adaptation*time |  | .01 | .01 | 102.24 | .92 |
| **Random effects** |  | **Variance** | **SE** |  | **Wald-Z** |
| Residual deviation |  | .17*** | .02 |  | 6.81 |
| Intercept deviation |  | .11*** | .03 |  | 3.59 |
| **p* < .05; ***p* < .01.; ****p* < .001 | | |  |  |  |

Table 7. Multilevel analysis of body esteem (appearance subscale) score by time and other implementation dimensions

| **Fixed effects** |  | **Coefficient** | **SE** | **df** | ***t*** |
| --- | --- | --- | --- | --- | --- |
| Intercept |  | 1.24*** | .07 | 155.64 | 18.84 |
| Adherence |  | .00 | .01 | 155.64 | .38 |
| Dosage |  | .01 | .01 | 155.64 | .78 |
| Acceptable adaptation |  | .03 | .02 | 155.64 | 1.66 |
| Unacceptable adaptation |  | .07* | .03 | 155.64 | 2.28 |
| *Level 1: intraindividual effects on intuitive eating* | |  |  |  |  |
| Time |  | .10*** | .01 | 99.21 | 7.29 |
| *Level 2: between participants effects on intuitive eating* | |  |  |  |  |
| Adherence*time |  | .00 | .00 | 99.65 | 1.29 |
| Dosage*time |  | .00 | .00 | 102.08 | .39 |
| Acceptable adaptation*time |  | .00 | .00 | 99.96 | .17 |
| Unacceptable adaptation*time |  | -.00 | .01 | 96.88 | -.25 |
| **Random effects** |  | **Variance** | **SE** |  | **Wald-Z** |
| Residual deviation |  | .15*** | .02 |  | 6.80 |
| Intercept deviation |  | .43*** | .7 |  | 6.54 |
| **p* < .05; ***p* < .01.; ****p* < .001 | | |  |  |  |
